# Challenges and opportunities in leveraging an existing systematic evidence database for mitigating hazards to the global food system

**DOI:** 10.1098/rsos.241645

**Published:** 2025-03-05

**Authors:** David Frederick Willer, Samuel W. Short, Diana Khripko, Julie Bremner, David C. Aldridge, William J. Sutherland, Silviu O. Petrovan

**Affiliations:** ^1^ Department of Zoology, University of Cambridge, The David Attenborough Building, Pembroke Street, Cambridge CB2 3QZ, UK; ^2^ IfM Engage, Institute for Manufacturing, University of Cambridge, 17 Charles Babbage Road, Cambridge CB3 0FS, UK; ^3^ Centre for Environment, Fisheries and Aquaculture Science, Pakefield Road, Lowestoft NR33 0HT, UK; ^4^ Collaborative Centre for Sustainable Use of the Seas, School of Environmental Sciences, University of East Anglia, Norwich Research Park, Norwich NR4 7TJ, UK

**Keywords:** agriculture, conservation, food security, sustainability, human-wildlife conflict, risk management

## Abstract

The global food system, essential for delivering nutritional security to a growing population, is highly vulnerable to diverse hazards. This study investigates the feasibility of leveraging an existing systematic database, specifically the Conservation Evidence database, for mitigating environmental hazards impacting the food system. By focusing on human–wildlife conflict as a case study, we explored the database’s potential to inform hazard mitigation strategies. Our analysis revealed significant geographical and taxonomic gaps, varied intervention strategies and differences in study designs across regions. We identified key challenges, such as the need for comprehensive tagging and filtering features, integration of non-academic data and broader stakeholder engagement. The findings underscore the complexity of adapting conservation databases for food system applications but highlight the potential benefits of a free-to-access, systematic, evidence-based approach focusing on food production hazard mitigation. Future work should focus on developing a dedicated food system hazard database, leveraging automation and machine learning to enhance data extraction and application efficacy, ultimately improving global food security and sustainability.

## 1. Introduction

Our global food system is the fundamental backbone of human civilization, bound to the task of delivering nutritional security to over 10 billion people by 2050 [1]. The system is also extremely complex, consisting of multi-step supply chains and logistics, diverse farming systems and products, deep integration with global economic and political networks and stakeholders and intractably connected to human culture and tradition [1,2]. The combined critical importance and overwhelming complexity of the food system makes it highly vulnerable to hazards, which can reverberate with global repercussions [3,4]. Hazards can emerge owing to high-level geopolitical activity cascading down food systems. For example, the global food crisis of 2007/2008, where a doubling in the FAO (Food and Agriculture Organisation of the United Nations) price index owing to high oil prices, low food reserves and export bans pushed over 100 million more people into hunger [5]. Hazards can also emerge from small-scale local issues, which when replicated across multiple locations create large and global tension. For example, while illegal fishing of a single species (*Arapaima* spp. where 77% of catch is illegal) in the Amazon lower basin might seem like a minor problem given the specificity, this behaviour repeats globally and contributes to the 30% of fishes that are caught illegally [6–9]. Hazards to the food system are thus numerous, diverse and complex, varying in their origin, intensity and differing across temporal and spatial scales [10].

As an illustration, a point of focus for this manuscript is human–wildlife conflict in food production settings, that is, negative interactions between wildlife and humans leading to adverse outcomes for either or both [11]. Human–wildlife conflict is often characterized by wildlife encroaching into farms and settlements, in the form of crop raiding and livestock predation, but it can more broadly encompass conflicts that emerge when the presence or behaviour of wildlife poses an actual or perceived, direct and recurring threat to human interests or needs. The competition between people and wildlife for food and resources is increasingly acute, especially in the Global South owing to agricultural expansion and the presence of large-bodied species such as elephants or other large mammals. Humans have long persecuted and locally eradicated species that were central in such conflicts, including large carnivores (e.g. in the UK, western Europe and parts of North America). This is exemplified by the fact that wild mammal populations have declined 85% since the rise of humans, primarily owing to hunting and agriculture [12]. Humans have also long worked on measures to minimize the negative interactions with wildlife, especially related to the protection of crops, livestock and guarding against attacks to human safety [13,14].

These measures are diverse, complex and often context-specific, including a broad range of social, behavioural, technical and physical approaches to reduce negative interactions with wildlife. Yet, the effectiveness of these measures is not always well described, and often there is a lack of a clear systemic approach to identify optimal measures [15]. Even when measures are described, they are often ineffective. For example, in Zimbabwe, where elephant–crop and lion–cattle conflicts are prevalent, annual household economic losses ranged from US$ 670 to 1000 between the years 2014 and 2016, despite the use of fires around fields at night and careful herding of livestock [16]. This is a major economic loss, with the average income per capita in Zimbabwe being just US$ 1345 in 2023 [17]. In this example, the use of electric fences might have been more effective, but practitioners lacked this information [16]. As it stands, our global capability to effectively identify and deploy hazard mitigation approaches is not yet sufficient, and a better ability to know, understand and implement appropriate food hazard mitigation approaches is critical in order to secure the health of our population, environment and biodiversity into the future [18,19].

An underpinning element behind the difficulties in identifying the most appropriate hazard mitigation is the availability of easily accessible evidence-based databases to inform decision making and best practices by practitioners. At present, to our knowledge, there is no single authoritative database which provides systematic and transparent access to the evidence on what hazard mitigation actions to take in order to tackle a single or multiple food system hazards. As a result, decisions are probably made on insufficient evidence, may suffer from complex biases, and effective decision making is generally hindered by the difficulty in accessing the evidence base [20]. Despite the large and increasing scientific and policy focus in evidence-based practice in recent years, many barriers remain, including communication and access issues, relevance (research often lacks direct use to practitioners or policy-makers), large untranslated primary data quantities, lack of comparative measures of effectiveness and costs and socio-political difficulties—changing the default pathway of action in conservation practice is not easy [21,22].

A logical step would be to systematically develop a comprehensive database on hazard mitigation in the food system. A systematic database for environmental hazard mitigation would provide an easy-to-work with source of transparently integrated and accessible data that could be customized via metadata such as precise location, local biome, hazard category, food sub-sector, to answer spatially explicit questions at different levels in the food system. This would highlight clearly where the evidence is weak or absent, either globally or regionally, and how different mitigating actions might compare to one another in terms of effectiveness, costs and certainty. Inclusion of studies in languages other than English is also important [23]. However, the realistic feasibility of building such a database from scratch is low—it would be extremely time and resource intensive to build and constantly update such a vast database given the literature base for food system hazards is large and rapidly increasing over time, with a 75% increase in publication rates over the past 20 years [24].

There may, however, be an opportunity to leverage systematic databases which already exist in other, somewhat related sectors for use in the food system. One example of such a systematic database, and the most comprehensive academic source relating to mitigation of environmental hazards in the biodiversity area, is the Conservation Evidence database [21]. The Conservation Evidence database gathers, organizes and summarizes studies that quantify the effects of conservation interventions on any aspect of biodiversity or human behaviour change related to biodiversity conservation. The database currently is based on the manual scanning of more than 1.6 million papers across 330 English-language journals and 325 non-English journals, with over 8600 studies now summarized. The database is split into subject areas, usually along taxonomic lines (e.g. bats, amphibians) with some taxa split by habitat (e.g. forest vegetation and shrubland vegetation). For each intervention, the database provides: background information such as the logic behind the intervention and how it might be carried out; standardized paragraphs summarizing individual scientific studies that have quantified the effects of that intervention; key messages that provide a narrative index to the combined evidence from all of those studies; and an overall effectiveness category based on an assessment of the evidence (effectiveness, certainty and harm) by a panel of experts [21]. The Conservation Evidence database uses expert panels to assign categories of effectiveness using a modified Delphi technique with anonymized scores and multiple rounds of scoring using standardized instructions where experts rank the evidence for each intervention based on 0 to 100 scores of effectiveness, certainty and harms. For users, these subject areas provide a rapid overview of the scope of the database, and filters to focus on the most relevant information depending on location, taxonomical level or type of intervention. In each subject area, the database provides a comprehensive list of interventions. The database has been built up over the past 20 years, applying a rigorous, transparent and structured approach (termed subject-wide evidence synthesis) to identification and documentation of the evidence, collating studies on intervention strategies and evidence of their application under specific actions clustered under International Union for the Conservation of Nature threats, regional deployment and relevance and effectiveness. The Conservation Evidence approach offers advantages over traditional literature reviews which are liable to bias and methodologically opaque [25] while systematic reviews, designed to reduce those issues, can be expensive, time-consuming and sometimes less suitable for topics such as biodiversity conservation where data are often scarce and study design and outputs are highly variable [26]. However, the Conservation Evidence database was not specifically designed for application to the food system and the mitigation of hazards in this sector.

In this study, we, therefore, aim to explore the viability of leveraging an existing systematic database, the Conservation Evidence database, for application to environmental hazards to the global food system. We aimed to gain an understanding of whether an existing database could be applied to the context of food system hazards and identifying mitigation strategies, the potential merits of using such an approach and the key challenges and areas for further work required to make this approach valid and useful. An in-depth case study of human–wildlife conflict was used to illustrate the approach and its limitations, owing to its global relevance to both food systems and conservation.

## 2. Material and methods

### 2.1. Hazard definition and identification

This study used the Centre for Environment, Fisheries and Aquaculture Science, UK (Cefas) and Animal and Plant Health Agency, UK (APHA) definition [27] for hazards to the food system as ‘any biological, chemical, physical factor acting on food production and distribution that prevents resources being turned into safely consumable food, or factors associated with food production that degrade the natural environment or contribute to climate change’ p. 1618. Note that by this definition, and therefore in this study, we focus on the ‘food production’ aspect of food systems, but do appreciate that other aspects of the food system such as transport, manufacturing and retail will have their own impacts, which we do not explicitly cover here. The Conservation Evidence database [28] was screened for the 29 environmental hazard categories previously defined in the [19] and [29] publications [19,29]. An initial top-level scan was performed by experts on the database, following a repeatable approach for identifying intervention and mitigation strategies across a range of the environmental hazards. This consisted of standardized searches across the database by topics and actions in order to relate the level of inclusion of different environmental hazards in the evidence base by topic or synopses. This informed the approach to data extraction, and to assess the scope for future studies across the entire database that could be leveraged for a food-system specific database. From the top-level scan, human–wildlife conflict was selected for deeper interrogation and analysis because, owing to its strong intersection with biodiversity conservation (the primary focus of the database), it is the most well-described hazard topic within Conservation Evidence, making it well-suited for the in-depth case study for this paper in terms of evidence availability in a systematically collated database. We also selected human–wildlife conflict on the grounds of its high relevance to food production, food security and sustainability. This is not to say that an assessment of other hazards would not be possible—harmful algal blooms are another example which could in future be explored via a case study in a similar fashion, and that does require more attention. We note that while the Conservation Evidence database coverage for human–wildlife conflict is relatively comprehensive, covering all major vertebrate groups and most major invertebrate groups, freshwater fish are not covered yet in the evidence synthesis and several of the synopses are in the process of updating.

### 2.2. Extraction of interventions from the Conservation Evidence database

**Figure 1.**
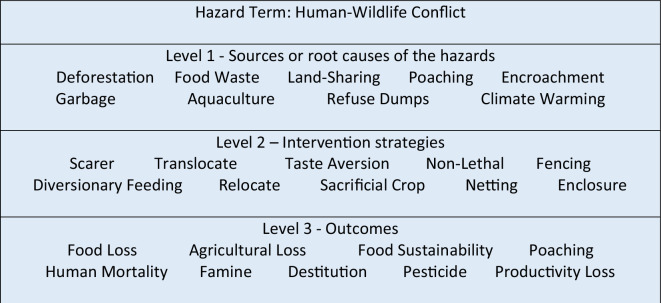
Search terms used to interrogate the Conservation Evidence database on human–wildlife conflict.

For the hazard of human–wildlife conflict, we started with a simple search term of ‘human–wildlife conflict’. However, human–wildlife conflict is not a separate topic for Conservation Evidence but rather draws actions and evidence from multiple separate synopses, and as such, this search only partially captured the relevant studies within the evidence database. Therefore, we systematically identified upstream root causes and downstream impact types and potential cascading hazards, and then identified the range of potential interventions or mitigation strategies for (i) prevention at source; (ii) mitigation and resilience building; and (iii) remediation of the end impact. This was used to support targeted literature searches, augmented with elicitation from the Conservation Evidence experts, to identify relevant search terms for each tier for the subsequent interrogation of the Conservation Evidence database. Based on this search, we built a schematic three-tiered set of Conservation Evidence search terms for human–wildlife conflict covering the sources or root causes of the hazards (level 1), intervention strategies (level 2) and potential negative outcomes of the hazard (level 3) (figure 1). The search terms were used to extract interventions from the Conservation Evidence database from the title of the intervention as well as the metadata and full intervention summary to generate data on total number of hazard interventions for each search term, total number of studies for each search term, a list of hazard interventions for each search term, alongside metadata covering geographical region, taxa and level of effectiveness or success where available. All resulting database interventions were manually screened for topic relevance and validation as there were many results that did not fall clearly in the human–wildlife conflict area but were rather about other, related hazards. In instances where taxa classifications were incomplete, for example, where only species but not family names were present, the software package Python [30] in combination with the Global Biodiversity Information Facility dataset API (Application Programming Interface) [31] allowed incomplete data to be identified and completed. Random spot-checks were then completed on a subset of 20 studies to ensure that interventions had been correctly tagged and classified.

### 2.3. Characterization of human–wildlife conflict case study

Using the extracted data from the Conservation Evidence database, we were able to calculate and explore the total number of studies relevant to human wildlife conflict and provide further characterization based on the metadata and summary information attached to each study and species entry. We provided characterization on the study design, the mitigation approach category, target taxa category and an insight on mitigation effectiveness, as presented in the results.

## 3. Results

### 3.1. Categories of mitigation and study type for human–wildlife conflict

Our review identified 158 studies and 593 associated mitigation strategies, which broadly fell into six categories for human–wildlife conflict. These specifically were: (i) audial, e.g. noise interventions to disturb birds, playing predator calls to deter herbivores from crop systems; (ii) olfactory, e.g. using predator scents, using conditioned taste aversion; (iii) physical, e.g. electric fencing, escape devices on fishing gear; (iv) regulatory, e.g. providing sacrificial grasslands to avoid herbivores eating crops, setting up voluntary agreement with locals; (v) relocation, e.g. translocating predators away from livestock, capture and release of problem wild animals; and (vi) visual, e.g. using decoys to attract birds to safe areas, using flags to deter herbivores. We also identified five key types of study: before and after studies, comparison studies, paired studies, replicated studies and single studies (i.e. data collected only after mitigation implementation). Overall, we were able to identify a total of 72 specific mitigation actions from a total of 525 instances of mitigation in studies in the Conservation Evidence database, most of them relevant for level 2 (figure 1), that span multiple subtopics and taxa groups. In addition to this, we identified 10 interventions (as created with the help of the advisory board for each synopsis based on subject-level knowledge) where we found no studies documenting the specific intervention. Most of these interventions (9 out of 10) were on terrestrial mammals and included audial interventions (e.g. play predator calls to deter crop damage by mammals to reduce human–wildlife conflict); olfactory (use pheromones to deter predation of livestock by mammals to reduce human–wildlife conflict); visual (i.e. use scarecrows to deter crop damage by mammals to reduce human–wildlife conflict); regulatory (e.g. carry out surveillance of bats to prevent the spread of disease/viruses to humans to reduce human–wildlife conflict); and physical interventions (e.g. use fencing/netting to reduce predation of fish stock by mammals to reduce human–wildlife conflict). These interventions were excluded from further analyses as they did not include any additional metadata information given that they contained no evidence.

### 3.2. Study designs by country

**Figure 2.**
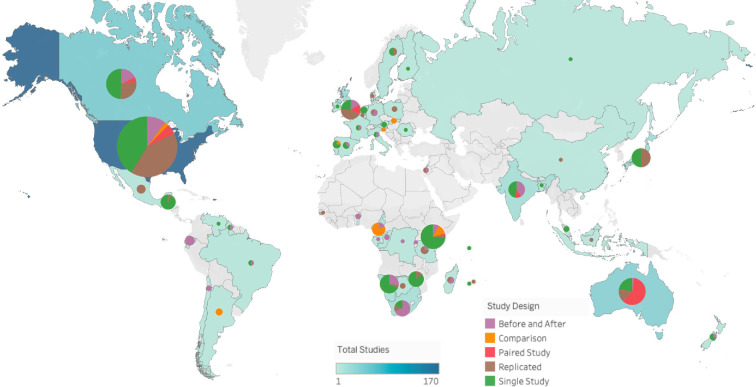
Study designs per country. The colour of each country and the size of their respective pie chart represent the total number of studies on human–wildlife conflict in the country. The colours on the pie charts show the proportion of these studies that fall under each study design category. The sizes of the pies are calibrated against the blue ‘Total Studies’ scale bar.

The majority of the studies for the human–wildlife conflict hazard were focused on the USA, followed by Canada and then other global regions including western Europe, Russia, Australia and small parts of southern Africa, South America and the Indian subcontinent (figure 2). There was a notable lack of studies from central and eastern Asia (e.g. China) and northern Africa—a major gap in the Conservation Evidence database. In the USA and Canada, studies were typically a mix of single studies and replicated studies, with a smaller proportion of before and after studies. In Africa, single studies were dominant, but there were also large proportions of paired studies, before and after studies and comparison studies. It is notable that in the UK, there were very few single studies (figure 2).

### 3.3. Mitigation categories by country

**Figure 3.**
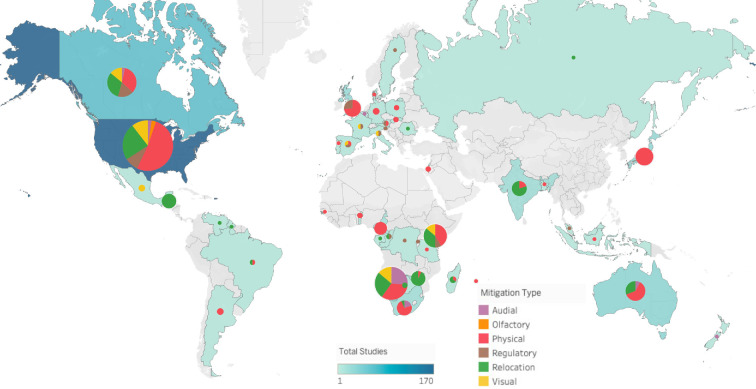
Mitigation categories by country. The colour of each country and the size of their respective pie chart represent the total number of studies on human–wildlife conflict in the country. The colours on the pie charts show the proportion of these studies that fall under each mitigation type.

Our analyses revealed strong differences in how human–wildlife conflict hazards were typically mitigated in different global regions (figure 3). Definitions for each mitigation category were outlined in §3.1. In the USA, Europe, Japan and Australia, physical interventions were dominant, making up over 50% of mitigations. In comparison, in Africa, there was a greater mix of interventions, with over 30% of interventions being relocations in eastern Africa, and over 25% of interventions being audial in southern Africa. In India, relocations made up over 75% of interventions, while in Europe relocation was a very uncommon mitigation approach.

### 3.4. Mitigations by taxa

**Figure 4.**
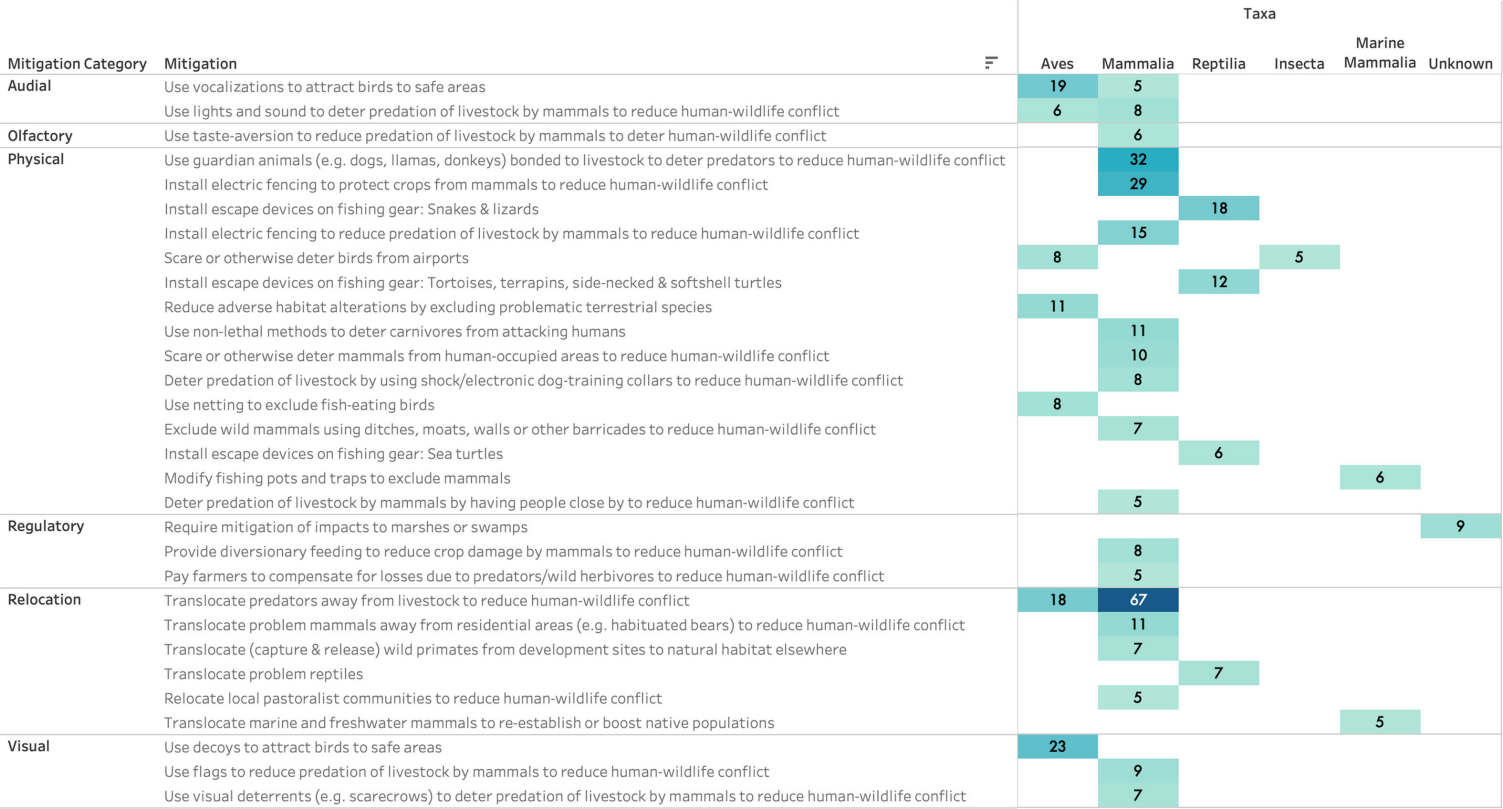
Mitigations per taxa. The figure shows the number of studies on each mitigation action, broken down according to the taxonomic group to which the study applied. Note that the data have been filtered to only show mitigations where there were at least five cases of use for a given taxon.

When assessing mitigations by taxa, the vast majority of human–wildlife conflict mitigations are targeted at terrestrial mammals, across all five of the mitigation type categories (figure 4). Translocation of predators away from livestock was the most common mitigation activity applied when trying to intervene on human–other mammal conflict (67 studies), followed by the use of guardian animals such as dogs (32 studies), and the installation of electric fencing (29 studies). We note that the majority of these mitigation actions targeted at terrestrial mammals occurred in Africa and North America (see the electronic supplementary material, ‘Collected Data’). After mammals, birds and reptiles were the next most common groups to which human–wildlife conflict mitigations were targeted. As for mammals, translocation of predators was important (18 studies), but of near equal importance were the use of visual decoys (23 studies) and the use of audial vocalizations (19 studies) to attract birds to safe areas and reduce conflicts. For human–reptile conflicts, installation of escape devices, for example on fishing gear, for snakes, lizards and turtles were common practice (30 studies in total). Bird conflict mitigations were particularly prominent in Europe and North America, whilst reptile mitigations were most prominent in Australia (electronic supplementary material, ‘Collected Data’). For marine mammals, modification of fishing methods and translocation were particularly frequent. Studies mentioning insects or invertebrates were numerous but were almost always excluded as the specific framing as human–wildlife conflict was difficult to establish and insect damage (e.g. locust plagues which are critically important for food production and security in Africa) tends to be treated simply as pest control, rather than conflict.

### 3.5. Mitigation effectiveness

It is possible to gain important understanding of hazard mitigation effectiveness using data from the Conservation Evidence database. The evidence assessments for the numerous actions listed under human–wildlife conflict varied widely, from ‘likely to be ineffective or harmful’ in the case of ‘translocating problem mammals (bears) from residential areas to mitigate human–wildlife conflict’ or ‘scaring birds from fish-farms’, to ‘trade-off between benefits and harms’ in the case of ‘translocating crop raiders or livestock predators’ or ‘likely to be beneficial’ in the case of ‘scaring or otherwise deterring mammals from human-occupied areas’. While some actions had very little or no evidence, others had a substantial evidence base, across several countries and many were assessed by the expert panel for each synopsis as ‘beneficial’. This ‘beneficial’ category includes several actions dealing with physical interventions such as installation of electric fencing to reduce crop or livestock predation or the provision of guarding animals such as dogs or llamas to protect livestock from predation in an effort to reduce human–wildlife conflict.

## 4. Discussion

Our study has revealed that there could be value in leveraging the Conservation Evidence database for food system hazard mitigation, if correct measures are taken to apply the database with appropriate improvements we discuss below. We acknowledge that the human–wildlife conflict case-study analysis revealed significant gaps and limitations, and that this database was not created specifically for food systems and thus other studies, which did not include a biodiversity outcome, were not included. Yet, we argue that this database remains of high utility to systematic food system hazard interrogation, enabling rapid access to an extensive array of methodically assessed intervention strategies, far more expediently and more comprehensively than known alternative search solutions to date. The conservation measures in the database and food system hazard intervention measures are similarly structured and closely matched, despite the fact that the conservation measures were not designed for food systems. The option of analysing the data and metadata using multiple relevant filters such as geographies, type of study and type of measure offers valuable, transparent approaches which are needed for addressing environmental hazards in the food sector. The Conservation Evidence database’s continually updated and structured approach makes it a stand-out example of how an effective systematic tool can function, and we suggest it offers an interesting template for a similar wide-ranging approach focusing on food system hazard mitigation.

### 4.1. Specific insights from the human–wildlife conflict case study

The case study analysis revealed several prominent features regarding the quality and validity of the Conservation Evidence dataset in its potential application to global food systems in the context of human–wildlife conflict. First, there are geographical gaps in the dataset—particularly for northern Africa and central and eastern Asia. This may stem from a true lack of data, but may also be a result of other threats or taxa being seen as of greater importance and thus receiving research attention instead of human–wildlife conflict [22]. We note that evidence is also not uniformly available across all intervention types despite the inclusion in the Conservation Evidence database of both peer-reviewed and grey literature (i.e. organizational reports) and non-English literature.

Second, there are disparities in the types of study available on a geographical level. In the Global South, there is a greater proportion of single or ‘after’ studies, which are far less reliable compared to the replicated, before-and-after or paired studies more prevalent in more research-intensive nations [32]. This may indeed reflect economic resource availability for research, with greater resources available in the Global North [33], but could also be an important indicator of the greater biodiversity, prevalence of unique ecosystems and lower levels of primary habitat disturbance in developing regions [34], and in fact highlight the damage that further human–wildlife conflict could cause in these regions. This is important, as there is already extensive evidence that the level of disturbance in these primary habitats in the Global South is increasing [35]. However, both of these aspects are also a reflection of wider biases and issues in the conservation literature, with low comparable coverage of the evidence in the Global South and particularly, in parts of Africa and Asia [36] as well as a decline in the robustness of study design in these regions [37]. Matching this theory, our analyses did reveal a greater diversity of intervention studies applicable to the Global South, which emphasizes how intervention strategies need to be sensitive to local contexts, cultures and environmental policies.

Third, our assessment shown in figure 4 provided important insight on how interventions are taxa-specific—for mammals, physical interventions are most prevalent, while audial and visual interventions have increased importance for birds, and solutions allowing escape are particularly important for reptiles—reflecting the critical importance of any hazard intervention database for food systems having broad coverage across all taxa interacting with the food system.

Finally, on a more general level, the case study demonstrated the importance and implications of correct search term selection in this form of analysis. A single-phrase search for ‘human–wildlife conflict’ on the Conservation Evidence database would yield about 90 intervention actions, but some of those were not specifically on human–wildlife conflict while many other relevant studies were excluded as they did not contain the exact terms in their description. Developing this set of terms required expertise, good understanding of hazards, potential solutions and outcomes, and valuable researcher time for manual verification of search outputs. For effective use in food systems, a database like Conservation Evidence would require more tailored metadata tagging and filtering features for studies so that evidence searches are best tailored to the specific demands of the sector. This would be best achieved by co-production of the database with a wide and diverse group of stakeholders from the sector.

### 4.2. Broader challenges and potential solutions for application of the Conservation Evidence database to food system hazards

On a broader level, our interaction with the database flagged several challenges to the application of the Conservation Evidence dataset to food system hazards, but also elucidated key steps that could need to be taken to make leverage of such a dataset to food systems a credible approach.

#### 4.2.1. Data-related challenges

Data quality is currently a challenge when leveraging Conservation Evidence to food systems. Many of the studies in the dataset are single studies, common for conservation research owing to the complexity of the studied systems, the diversity of measured outcomes and the substantial difficulties in collecting multiple seasons multiple sites data on wildlife species that have low density or high mobility. The use of less robust study designs (i.e. without replication, comparison, paired designs or before-and-after assessment) is particularly relevant in the Global South, where conservation resources are often stretched and make such implementations difficult. Food systems data are more commonly found in more robust formats, given humans tend to repeat the same food production practices on a global level and there is a smaller range of food system types than there are species requiring conservation [38]. This data on food systems does exist—but most of it is not in the Conservation Evidence database, and there would be a need for this type of data to be integrated to create a more robust food system database.

The Conservation Evidence database also lacks specific data types which are needed for investigating some hazards. For example, the database lacks metadata on whether interventions are preventative, mitigation and resilience building or remedial. Some interventions such as community engagement, regulatory interventions or early warning systems are also poorly covered in the database. Such items are typically missing as they are not specifically about implementing a conservation action and also as they are rarely monitored with a biodiversity outcome. Additionally, data on resources required, costs of implementation and cost–benefit analysis of interventions are typically not reported in the peer-reviewed literature for conservation, are not standardized [39], and are not separately captured as metadata. All of the aforementioned data types are, however, highly relevant to food systems hazards. For a systematic database to be truly applicable to food systems and offer guidance for practitioners, it will need to make intense use of data from non-academic sources—for example, industry reports to provide more information on costings, and regulatory and government reports which often provide more data on whether interventions are preventative, resilience building or remedial. This data will be fragmented and often difficult to access or not even captured in formally published reports. There are some quality data sources such as the FAO’s legislative and policy index, FAOLEX [40] and FAO’s AGRIS International System for Agricultural Science and Technology [41], which could be mined in a standardized and repeatable manner. Access to less publicly available sources will probably require close collaboration with key industry players, and for any proprietary data, agreements on collaboration and sharing of data will be necessary.

#### 4.2.2. Automation and analytics capabilities and requirements

For the Conservation Evidence database to be more universally applied to food systems, there will be a need for further automation. To date, the database has been painstakingly built up over two decades with many thousands of person-hours of work assessing academic papers one-by-one. This process provides a quality output but is not easily scalable and especially for an even larger sector such as food production hazard mitigation. However, extensive progress is being made, using evolving artificial intelligence (AI) tools that could make the integration of new datasets into conservation evidence or the creation of completely new databases for new fields, like food systems [42,43], a task that takes a fraction of the time as for the original database. To provide context, one example is the use of retrieval-augmented generation (RAG) machine-learning approaches to facilitate semi-automation of literature screening, data and meta-data extraction, customization of tools and decision-making [44]. RAG machine learning aims to build a ‘copilot’, i.e. a trained chat interface that can be interrogated to provide practitioners access to state-of-the-art data on relevant intervention strategies for any given scenario, at a high level of speed and cost efficiency. This process supports and increases the use of a critical element of the database; the direct and transparent linking of the studies within each intervention to the underlying information source. This element allows practitioners to select actions and then check the exact evidence context, strength and effectiveness by referring to the assessment category for each action, and also, if required, to access the full publication and check additional contextual or methodological details. This then also facilitates the customization of ‘evidence blocks’ across any filtering depending on species of interest, type of action, habitat or geographical location.

#### 4.2.3. Technical system architecture

There are key architectural elements of the Conservation Evidence database which are not fully compatible with food system hazards, and these require adjustments and considerations to be made for application to food systems. One key pinch-point is the lack of parsimonious approaches to tag interventions when they address multiple hazards. Additional information on relationships between hazards needs to be included in the database. This may be difficult; studies are often narrow, extrapolation to other geographical or climatic contexts is often limited, or hazards and interventions may be studied in isolation, providing limited insight into interactions between hazards. There is a need for extraction of additional metadata from studies that could support the development of complex intervention network maps that characterize hazard interactions.

An element of system architecture that is critical for an effective food system mitigation tool is a powerful, easy-to-use front end for practitioners. There is a need for an open data platform, for example, similar to European data [45], where one can use the interface to customize and visualize the data but also download relevant parts of statistics or other outputs.

#### 4.2.4. Development challenges

There will also be fundamental development challenges at a socioeconomic and political level when trying to repurpose and adapt existing systematic databases like Conservation Evidence for food system hazards. Stakeholders are different and more diverse for food systems. The development of the Conservation Evidence database required engagement with a range of stakeholders, but given the primary scope of the database, was generally restricted to experts from ecology and conservation. However, the food system involves a broader and more complex array of factors compared to ecological conservation. It includes substantially different interactions between environmental, economic, social and health dimensions, and therefore development of a similar evidence database for food system hazards will necessitate a much broader coalition of participants or stakeholders both for collation of data and use of the database. These stakeholders will need to include the farming communities at large and small scales, industry, municipalities, national government and third-sector and multilateral organizations, alongside academic researchers. There will also be the logistical need to clearly coordinate functions for initiation, development and operation of such a database.

## 5. Conclusion

Leveraging existing large-scale systematic datasets of evidence, like the Conservation Evidence database, presents a significant opportunity for addressing global food system hazards—if key technical challenges are effectively tackled. These forms of database offer a structured approach to synthesizing diverse intervention strategies, providing rapid access to rigorously assessed evidence that can guide hazard mitigation. However, to maximize use in the food sector, enhancements are needed, such as improved tagging, integration of non-academic data from industry or regulatory sources and the development of user-friendly interfaces. Addressing geographical and taxonomic gaps and ensuring the inclusion of comprehensive metadata on intervention effectiveness and costs are crucial steps. By harnessing advances in automation and analytics, alongside fostering collaborative efforts across stakeholders, there is an opportunity to develop a robust, adaptable tool capable of supporting evidence-based decision-making in the food system. Such a tool would not only strengthen global food security and sustainability but also pave the way for a more resilient food system capable of withstanding complex environmental challenges, and one which reduces the impact of agriculture on wildlife and biodiversity.

Further investigation of this and other evidence databases as well as structured stakeholder engagement events are recommended to lay the foundations for developing a full food system hazard intervention database. Researchers could explore the evolution of evidence databases to date, the challenges and lessons learnt, further explore the application of AI and ML (Machine Learning) to high-quality and human-verified evidence synthesis, how other existing databases might be leveraged, and importantly explore how best to engage stakeholders to ensure enduring value of the database.

## Data Availability

All data are available in the manuscript or electronic supplementary material [46].
